# Deliberation in Guesstimation

**DOI:** 10.1111/cogs.70090

**Published:** 2025-08-13

**Authors:** Vildan Salikutluk, Frank Jäkel

**Affiliations:** ^1^ Centre for Cognitive Science Technical University of Darmstadt

**Keywords:** Guesstimation, Deliberation, Judgments, Problem‐solving, Mixed‐methods, Human experimentation

## Abstract

In many real‐world settings, people often have to make judgments with incomplete information. Estimating unknown quantities without using precise quantitative modeling and data is called guesstimation, which is often needed in forecasting settings. Furthermore, research in education found that solving guesstimation problems builds general problem‐solving skills. In this paper, we present an empirical investigation on how people solve guesstimation problems. We study their problem‐solving behavior with think‐aloud methods, and we identify solution strategies that are frequently used. In a two‐response paradigm, we first ask for gut‐feeling answers to guesstimation questions and then allow deliberation before a second answer is given. Comparing the quality of these two answers reveals that deliberation improves the answer quality significantly. In a second experiment, we additionally elicit participants' confidence about their deliberated answers by asking for an entire distribution instead of just a point estimate. We find that participants are generally overconfident in their answers. We discuss guesstimation tasks as suitable test‐beds for studying human deliberative judgments in general and in the more specific context of improving forecasting through appropriate artificial intelligence tools.

## 1 Introduction

In many real‐world forecasting sscenarios, analysts need to make difficult judgments with incomplete information (Tetlock & Gardner, 2015). Such settings require them to produce forecasts under extreme uncertainty, for example, when drafting a business plan and calculating the demand of a product (Anderson & Sherman, 2010; Fildes, Ma, & Kolassa, 2022), when assessing health risks (Bertozzi, Franco, Mohler, Short, & Sledge, 2020; Petropoulos et al., 2022), or when making (geo‐)political judgments or predictions (Mellers et al., 2015; Tetlock & Gardner, 2015). There are many cases where precise quantitative modeling is not an option or relevant data is simply not available. A recent example is the beginning of the COVID‐19 pandemic, where scientists did not have reliable data on how infectious and deadly this new disease really was, but politicians still had to make high‐stakes decisions based on rough estimates. Estimating unknown quantities from incomplete or highly uncertain information is called *guesstimation*. To produce the best possible answers in such situations, deliberating different options, strategies, and approaches is crucial (Haran, Ritov, & Mellers, 2013; Tetlock & Gardner, 2015).

Guesstimation problems are also called *Fermi problems* because the physicist Enrico Fermi was famous for posing such (theoretical) problems in class, for example, “How many piano tuners are there in Chicago?” (Weinstein & Adam, 2008). Unless students can directly google the answer (which they could not in Fermi's time), they had to find creative solutions by decomposing the question into subquestions that they could answer. One solution strategy for the example question is to get estimates for “How many pianos are there in Chicago?” and “How many customers does a piano tuner have?”. By dividing the former by the latter, one can compute an answer. However, both of these questions can probably only be answered by decomposing them again into further subquestions, like “How often does a piano need tuning?” and “How long does it take to tune a piano?”, and so on, until all subquestions can be answered.

Not only can such back‐of‐the‐envelope calculations provide good estimates, several studies also demonstrate that learning to solve them has positive effect on critical thinking skills and creativity (Ärlebäck & Albarracín, 2019b; Holubova, 2017; Hartmann, Borys, Kawasaki, & Okamoto, 2019; Okamoto, 2022).

Most tasks in school require pupils to only apply one modeling cycle, that is, analyze a given problem in order to understand what is asked of them, find and execute the appropriate calculations, and give the final answer. However, guesstimation problems can be used as *model‐eliciting‐tasks*: Pupils have to devise solution plans (Albarracín & Gorgorió, 2014) and answer multiple subquestions, which, in turn, require multiple cycles of mathematical modeling (Peter‐Koop, 2004). Tackling guesstimation problems thus improves students' general problem‐solving skills and performance in math classes across different ages (Albarracín & Gorgorió, 2014, 2015), and fosters skills required for all STEM subjects (Ärlebäck & Albarracín, 2019b). Furthermore, the ability to give reasonable answers to guesstimation questions can serve as an indicator for a person's mental flexibility, creativity, and quantitative abilities, which is why they are often used during job interviews and in assessments centers (Anderson & Sherman, 2010; Weinstein, 2012; Wessels, 2014).

Even though there are studies on testing and cultivating the forecasting capabilities of experts (Mellers et al., 2015) and best‐practice guides on guesstimation (Swartz, 2003; Weinstein & Adam, 2008; Weinstein, 2012), there is a lack of empirical work on the underlying cognitive solution process and potential impasses that might arise. Given the practical importance of such guesstimation problems for many real‐world decisions as well as their prospect for teaching students crucial problem‐solving skills for the 21st century (Ärlebäck & Albarracín, 2019b), we investigated *how* people answer guesstimation questions.

Also, in most previous studies, the answer is a probability for a binary event (Mellers et al., 2015) rather than a real number. Even in studies in which real numbers are elicited, the questions used would be easy to answer if participants had access to the internet (Gomilsek, Hoffrage, & Marewski, 2024). Having access to the internet, however, is an arguably more plausible scenario for guesstimation tasks in the real world. Therefore, we conduct experiments representative of a realistic guesstimation setting with access to the internet to examine how people perform and what is required to solve such problems successfully.

### 1.1 Strategies to answer guesstimation questions

In previous work, different approaches to study human guesstimation have been developed. Ärlebäck and Albarracín (2019a) proposed extending Model Activity Diagrams (MADs) to study guesstimation in pupils. They divide the process into six activities, consisting of reading, modeling, estimating, calculating, validating, and writing, which they use to generate a graphical representation of the activities and when a student engages in them. Another approach to study human guesstimation are “Fermi‐Trees” (Hartmann, Borys, Okamoto, & Kawasaki, 2020). They are also used to model the steps and calculations pupils take within a guesstimation process. The steps modeled in these Fermi‐trees are similar to those in MADs, but they are shown in the temporal order, that is, in the order that the students applied the steps (including inconsequential steps or errors). This approach was also used in research about creativity in guesstimation (Okamoto, 2022). All the aforementioned approaches were developed as didactic tools and to understand the guesstimation process of pupils in mathematics classes. While these studies produce valuable insights, they do not evaluate the accuracy of the estimates. Furthermore, they mostly focus on the specific calculations of the students and how they report their results, not on general strategies to decompose or transform the questions to find the best possible answers.

In contrast, Paritosh and Forbus (2004) identified and formalized different strategies for both the decomposition and solution of guesstimation problems. They used these strategies to implement the BotE‐Solver (Back‐of‐the‐Envelop‐Solver), a system that can answer guesstimation questions “in the right ballpark,” that is, its answers are not off by more than one order of magnitude for a small set of test questions (eight questions in a first paper (Paritosh & Forbus, 2004) and 13 in a follow‐up paper (Paritosh & Forbus, 2005)). Another such system is GORT (Guesstimation with Ontologies and Reasoning Techniques) by Abourbih, Bundy, & McNeill (2010). GORT is a semi‐automated system that combines semantic web technology with planning and reasoning methods, which are used to decompose guesstimation questions and try to answer them. If GORT can fully decompose a question and find the answers to all subquestions, it can answer the question by itself. However, if GORT is unable to further decompose the questions with the implemented methods, it asks a human for a guess or an answer for the question. While its methods are not exhaustive and some are domain‐specific, they probably still capture some aspects of human problem‐solving because they were based on a popular best‐practice guide for guesstimation (Weinstein & Adam, 2008; Weinstein, 2012). Some methods used for GORT and the BotE‐Solver that are applicable in general and are used in the evaluation of our first experiment below are the following:

- **Average Value**: Strategy to calculate the average value for a certain aspect of a question, for example, “What is the average runtime of a typical film?” which is then calculated based on knowledge about runtimes of a set of known films.
- **Aggregation over Parts**: This strategy is applicable if an object is decomposable into smaller, distinct, and nonoverlapping parts. The strategy is to find estimates for the parts and combine them into the value for the original object, for example, to calculate the population of a continent (object of interest), you need to add the population of all countries within it (nonoverlapping parts).
- **Size Plan**: Strategy to calculate the size of a number of objects, for example, “What area would be required if all humans in the world were put in one place?” If the value for the area that a human would occupy is known (or estimated), and the overall number of humans in the world is known as well, the required area and its value can be calculated. As opposed to the *Aggregation over Parts* strategy, the smaller parts of the object of interest (area that would be required for every human) are equivalent (an average size for a human occupying space could be used here).
- **Scale Unit Conversion**: Transform the unit of a number into another which can be used in the calculation, for example, from kilometers to meters.
- **Density**: Strategy to convert a quantity into a density quantity and an extent quantity (Paritosh and Forbus, 2005). Density refers to an average along any dimension and, generally, can be used to solve problems that can be represented as ratios. A special case of the density strategy is the use of proportions. Calculating proportions and using percentage rules was the main way our participants in Experiment 1 used this strategy, thus, we refer to this strategy as “proportion” throughout. For example, participants used this strategy when answering questions such as “What proportion of people in Brazil are young?” by determining the number of people living in Brazil and estimating how many of them are young (setting a certain threshold of age) to then calculate a proportion from these two numbers.

The semi‐automated approach of GORT and the BotE‐Solver were successfully solving a small set of test questions and both employ reasonable approaches and strategies (Paritosh & Forbus, 2013), that are, for example, derived from books that describe best practices and example solutions (Swartz, 2003; Weinstein & Adam, 2008). However, since the set of questions answered with them were limited, it is likely that these strategies are not exhaustive with respect to the approaches that people use to solve guesstimation problems across several different domains.

### 1.2 Uncertainty and deliberation in guesstimation

In recent work, Gomilsek et al. (2024) demonstrate that participants improve upon their accuracy from a first estimate to a second one, if for the second estimate, they receive instructions that encourage deliberation about the decomposition of a guesstimation task. They also show that such deliberation works better than other strategies, such as considering the first answer to be wrong and then estimating again. In other studies, where group and individual answers are compared in forecasting or estimation tasks (Silver, Mellers, & Tetlock, 2021), group deliberation can have a positive influence on answer quality compared to individual answers, but only when group members are well‐calibrated, that is, more knowledgeable members are also more confident, and contribute to the group answer more than the less confident members (Mellers et al., 2015, 2015; Silver et al., 2021). Such improvement through group deliberation can also be found for estimation tasks when knowledge within a group is transferred to its less informed members (Schultze, Mojzisch, & Schulz‐Hardt, 2012). Just like in many other tasks (reviewed in, e.g., Chabris & Simons, 2009), overconfidence is also an issue in guesstimation‐like tasks (Gomilsek et al., 2024).

These studies show that deliberation can have a positive effect on answer quality in guesstimation‐like tasks, but also that being well‐calibrated about one's answers is crucial, too (Bennett, Benjamin, Mistry, & Steyvers, 2018). Therefore, in this paper, we do not only study how and how well people solve guesstimation problems, but also investigate whether their reported certainty about their judgments are well‐calibrated.

### 1.3 Overview of experiments

We empirically investigate how humans approach and answer guesstimation questions with two experiments. In Experiment 1, participants were instructed to solve guesstimation problems while thinking aloud. Based on the think‐aloud protocols, we reconstructed and formalized how they compute their solutions and identify some crucial aspects of successful solutions. As expected, participants decomposed questions into subquestions, but they also often replaced questions that they could not answer with semantically related ones that they felt were easier to answer. The empirically identified strategies align with previous theoretical work (e.g., Paritosh & Forbus, 2004; Paritosh and Forbus, 2005; Abourbih, 2009; Abourbih et al., 2010) but also go beyond them. In our study, participants had to first give an intuitive answer and then provided a second response after (extensive) deliberation. We find that performance increases through deliberation. Furthermore, in Experiment 2, we study how sure participants were about their final answers and analyzed not only their performance but also asked them to provide a certainty judgment. Similar to the study by Gomilsek et al. (2024), this allowed us to investigate the calibration of our participants about their given answers. In contrast to the study by Gomilsek et al. (2024), we designed an experimental setup that did not use questions that would be easy to answer with access to the internet. In our study, participants could use the internet to find relevant information, but we made sure that the correct answers were not directly accessible, either because they were behind pay‐walls or were based on unpublished data that we, however, had access to.

## 2 Experiment 1: Solving guesstimation problems

In our first study, we ask participants to think aloud while they solve guesstimation problems. We examine which steps and strategies are necessary for such tasks and what the underlying solution process looks like. Furthermore, we elicit gut‐feeling and deliberated answers and examine the difference between them.

### 2.1 Methods

We conducted a think‐aloud study with 10 participants who were all university students between the age of 20 and 24. They received partial course credit for their participation. The study was approved by the local ethics board and all participants provided informed consent. The study was conducted in their native language (German) and in a lab environment. The participants were asked to solve six guesstimation problems and think aloud while doing so. Participants were first tasked with solving one training question (“How many rice kernels fit into a coffee mug?”) to get acquainted with the interface shown in A.2 in Fig. 5. This familiarization round also gave them a chance to think aloud and get used to sharing their thoughts. During the training round, as well as during all experimental trials, participants were regularly reminded to talk and share their thoughts whenever they stopped for around 20 seconds. The full and detailed instructions can be found in the Appendix (see A.1). The questions were chosen to cover a wide range of different domains. All guesstimation questions used in our experiments can be found in Table 1. We used a two‐response paradigm for each trial. In the first part, the participants were asked to give a quick “gut‐feeling” response within 30 seconds. For this first response, they were simply asked to put in a number they think is the correct answer. Once they answered with their gut‐feeling, they were asked to deliberate on the same question in the second part of each trial.

**Table 1:** All the guesstimation questions used in the experiments

**Guesstimation questions**		**Experiment**
1	How many car sharing vehicles are there in Darmstadt?	2
2	How much revenue was made in Germany since 2016 with the sale of musical instruments?	2
3	How many people in Brazil use music streaming services?	2
4	How many publications do all living Nobel Prize laureates in Economic Sciences have overall?	1 and 2
5	For all actresses who won an Oscar for Best Actress, how many movies did they act in, overall?	1 and 2
6	How many smartphone users are there in Dhaka?	1 and 2
7	How many minutes of TV were watched per person in 2020 in Belarus?	1
8	How much money did Spotify invest in Research and Development since 2018 (in €)?	1
9	How many student applications were sent to the Technical University of Darmstadt from Bavaria in 2022 (summer‐ and winter semester)?	1

*Note*. Please note that we made sure that the answers to the questions were not directly available online during the time the experiments were conducted. It is possible that this can change at any point. The answers for some of the questions became available after Experiment 1 and before Experiment 2, which is why those questions were replaced, and thus, some of them are only used in one of the experiments.

In the second part of each trial, participants had 8 minutes to provide the best answer they could, that is, an estimate as close to the unknown true value as possible (but we did not specify a loss function). They were allowed to research anything they wanted on Google, take notes, and use a calculator; all via a simple web interface that we provided, which is shown in A.2 in Fig. 5. When they were ready, they entered their answers into a dedicated field and proceeded to the next trial. During the experiment, we recorded a screen capture video, the think‐aloud audio data, the search terms and phrases for Google, and their notes and calculations. Our primary interest in this study was to find out how participants compute their best guess by decomposing a question into subquestions. Therefore, we ensured that the answers could not be found directly through Google. Participants thus had to decompose the questions into subquestions for which they could find the answers from relevant data on the internet or to estimate them (from experience).

Lastly, we asked the participants to fill out a questionnaire at the end of the experiment. They indicated their agreement to eight statements on a 5‐point Likert scale. These items were aimed at investigating several aspects, such as the participant's perception of their answer quality for both the gut‐feeling and deliberated answers. In addition, we asked about whether they were sure about their approaches to the questions and whether they would have liked additional help (in the form of tips or tools to use).

**Fig. 1:**
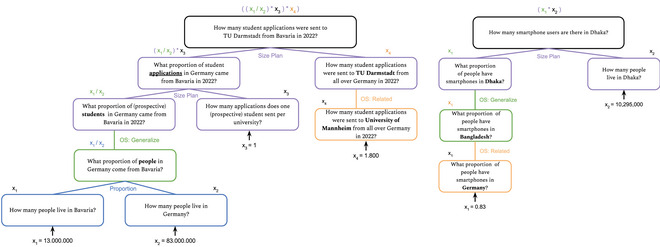
Examples for computation trees showing problem solutions for two questions in the think‐aloud study. If a tree is read bottom‐up, it shows which calculations and combinations of subanswers were necessary for the final answer. If it is read top‐down, it shows the decomposition of the original question, that is, the plan the participant followed. Each node in the tree shows a question and a variable, for example, $x_{1}$, for the value of the participant's response. Numbers are combined according to the rule that is applied, for example, calculating proportions. The values at the leaves represent either the information that participants found on the internet or the values they guessed or filled in from their knowledge. The concepts shown in boldface are those that were transformed during the solution process with the corresponding strategies.

The video and audio data were transcribed, and the resulting protocols containing all utterances of participants' thoughts were used to reconstruct how they computed their answers. Such think‐aloud protocols can be sparse, but we were able to match participants' search terms, their notes, and calculations with their uttered thoughts. In this way, we usually could reconstruct how participants computed their answers. We coded the protocols with a thematic analysis (Gibbs, 2007; Williams & Moser, 2019), which is used to identify common themes, that is, approaches, steps, and patterns that come up repeatedly. As the transcriptions included not only utterances but also video and behavioral data, such as searching on the web and using a calculator, we could often reconstruct the whole computation, even if participants did not explicitly utter every step (for examples of complete computation trees, see Fig. 1). Two raters coded the entire protocols independently. However, as reconstructing the computation trees requires not only coding of utterances but also inferences from observed behavior (web‐searches and calculations), we refrain from reporting a measure of reliability. Nevertheless, the two raters mostly agreed on how to classify the strategies and resolved any uncertainties about the data and its coding. It is important to note that we only reconstructed computations where the steps were clear from the data and refrained from assigning strategies for (parts of) answers where the process was not obvious or too much interpretation would have been necessary.

### 2.2 Results

Overall, participants were able to solve the guesstimation problems: For the 60 trials, we collected (six for each of the 10 participants) only one remained completely unanswered, that is, both the first (gut‐feeling response) and second part (deliberated answer) were not answered. In another four trials, a deliberated answer was not given because time ran out before participants could generate an answer. Of the remaining 55 trials, 17 trials were answered with pure guesses even after deliberation. Another six trials showed that participants researched and deliberated one part of the question and guessed another. This was the case, for example, for the question “How many smartphone users are there in Dhaka?”. Participants researched how many people live in Dhaka but guessed the proportion of those people who might have a smartphone (e.g., 80%). Then, they calculated their final answers with the corresponding numbers. While this is not a complete guess for their final answers, it is clear that a crucial part of it was simply guessed instead of decomposing or transforming this part of the question further to attain a better estimate. On average, participants completed a trial and gave their deliberated answers in 6 min and 21 s (mean = 381 s, *SD* = 68 s). The distribution over all participants' completion times is shown in the Appendix (see A.3 Fig. 6a).

#### 2.2.1 Analysis of solution strategies for guesstimation problems

We formalized the participants' solution steps for their answers to a given question as computation trees. Each tree shows the entire successful decomposition of a participant for a specific question and its (reconstructed) subquestions, as well as the corresponding necessary calculations. Note that not all answers of the participants were successful, and thus, not all of them can be formalized as such a tree. However, two examples of computation trees are shown in Fig. 1. If the trees are read top‐down, they show the decomposition of the original question, that is, the plan the participant followed. Each node in a tree shows a question and variable for a number as the participant's (sometimes implicit) response. Numbers are combined according to the rule that is applied. The leaves represent values, that is, explicit numbers, that participants found on the internet, guessed or filled in from their experience or knowledge. The concepts that are shown in boldface are ones that were transformed, that is, replaced with other concepts, during the solution process with the corresponding strategies (see “ontological similarity” below).

We coded the protocols with the strategies from GORT (Abourbih, 2009) as listed in the introduction. They are also shown in rows 1–4 in Table 2.

**Table 2:** Strategies identified in the data of Experiment 1

Strategy		Frequency
1	Size Plan	19
2	Aggregation Over Parts	13
3	Scale Unit Conversion	18
4	Average Value	17
5	Proportion	41
6	Ontological Similarity	68
7	Fudge Factors	19

*Note*. These strategies were used to code the data and describe the approaches used during the solution process of guesstimation problems. Their frequency, that is, how often they were used by the participants across all trials, is shown on the right. Note that the frequencies include all applications of the strategies, not just those from the successful solution approaches.

Although we were able to code a sizable part of the protocols with just these strategies, not every step was covered by them and, hence, we also derived codes from some of the strategies that are used in the BotE‐Solver (Paritosh and Forbus, 2005). However, we had to refine these original codes to better capture the protocol data. For example, we specialized the density‐strategy (that was already mentioned in the introduction) to a proportion‐strategy (row 5 in Table 2) because we did not see any evidence in their utterances that participants were thinking about densities. In another case, we combined two of the strategies of the BotE‐Solver into one:

- **Ontological similarity**: This strategy does not decompose a question into subquestions, but rather replaces a question with another one that is easier to answer (i.e., equate one value to another). This is done by changing or replacing at least one concept in the question to a related one. We found that participants choose such replacements in one of three ways. One such way is that they generalize a concept (e.g., Portuguese citizens to Europeans), that is, they moved up in the ontology. Another way participants replaced a concept is to specialize it or chose an instance of the concept (e.g., limousine to limousine of a specific brand X), meaning that they moved down in the ontology. Alternatively, they transformed a concept into a related one (e.g., Portuguese citizens to German citizens), which means they ascended in the ontology first (generalizing Portuguese citizens to Europeans) and then descended again (another instance of Europeans is, e.g., German citizens). This strategy was applied 68 times across all trials and is a combination of the “similarity” and the “ontology” strategy that are central to the BotE‐Solver (Paritosh and Forbus, 2005), hence, the name. While this strategy in its parts was previously identified, it is important here that we observed in our think‐aloud protocols that participants always applied them *together* during guesstimation. They usually do not explicitly state one step or strategy first, followed by the other. They take them as one unit. Therefore, we address these strategies and their application as one combined step.

On top of coding the strategies that were previously used in GORT and BotE‐Solver, we introduced one more code to be able to cover more utterances in the protocols:

- **Fudge factors**: Applying a factor to either increase or decrease a certain value. Importantly, this is not just a guess but the adjustment of a deliberated (partial) answer. However, based on the participants' intuitions, they adjust their estimate by a certain factor. There is not always a clear reason for why participants adjust values, but sometimes it is to ease calculations (rounding numbers up or down), or because they just “felt like” the number was too high or not high enough. For instance, a participant generated an estimate for how many movies an Oscar winning actress acts in during her entire lifetime. An average value of three movies was calculated per year and a duration of 40 years of working was guessed by the participant, which means that the average number of movies in such an actresses' career should be 120 based on the participant's calculations. However, this number seemed too high to the participant, and was, therefore, reduced to 100. Another participant calculated the overall number of movies all Oscar winners for Best Actress were in. While doing so, the participant remarked that some of those movies feature more than one of these actresses and reduced the calculated number of movies by 275 (from 1375 to 1100). While this difference of 275 was not explained specifically, the participant considered a specific information (more than one Oscar winning actress in the same movie) and adjusted the answer to include this information irrespective of checking whether this number itself was correct or probable. In our data, such fudge factors were used 19 times across all trials. In particular, participants used it to increase a deliberated (partial) answer eight times and reduce it 11 times.

Table 2 shows how often all strategies were used across all 60 trials and all participants. Almost all strategies directly related to the calculations that the participants performed, which can often be visualized as computations trees such as the examples shown in Fig. 1. However, note that we counted the strategies overall each time they were used, not just for the successful trials, that is, when they “completed a whole tree.” Furthermore, the applicability of the different strategies for calculations depends on the specific (sub)question at hand. Therefore, the frequency of usage varies from question to question as well as overall. Ontological similarity, which does not correspond to a calculation, was necessary in all trials and often used more than once throughout the calculations for an answer. Thus, participants often replaced a (sub)question with a related one that they could answer. Such substitutions were often made without mentioning them explicitly, but we could infer them from the participants' behavior, that is, their notes, the search terms, and calculations. Fig. 1 shows two examples of this: in the right‐hand branch of the tree on the left, the participant was unable to find an answer to the question they actually wanted to answer, that is, “How many student applications were sent to the Technical University of Darmstadt from all over Germany in 2022?”. They then replaced the question with “How many student applications were sent to University of Mannheim from all over Germany in 2022?”, meaning that they could not find the information for the university they were researching. However, they were able to find this information for another, comparable university. Thus, they used this value instead. While the two questions are likely to have different answers, the answer to the second question was easily accessible online and the participant simply used it to answer the original question. Another specific example of ontological similarity in the tree on the left is the question “What proportion of (prospective) students in Germany come from Bavaria?” which was replaced with “What proportion of people in Germany came from Bavaria?”. In the tree on the right in Fig. 1, the whole left branch of the tree shows how this strategy (in its different variations, i.e., generalized, specified, or related) is sometimes repeatedly and consecutively applied.

In addition to the strategies reported in Table 2, we also observed an interesting “meta‐strategy,” which we call *exploratory information search*. This refers to an exploratory behavior we often observed in the protocols. If the participants did not know how to answer a question at all, or they did not have any context for the content of the question, they would explore some facts about it first before being able to apply a strategy to solve it. Thus, exploratory information search also does not correspond to a calculation either. In comparison to ontological similarity, it does not even appear in the computation trees. It is rather aimed at finding a way to construct such a tree, that is, find a solution approach, in the first place. For example, one participant was trying to estimate how many movies an actress who won an Oscar for Best Actress would be in. To this end, the participant first researched how long an average acting career lasts (and found the number 45 years). The participant used this information as a first starting point to determine how many movies actresses are in during their entire career. Another example was the following: for the question “How many university applications were sent to the Technical University of Darmstadt from Bavaria in 2022 (summer‐ and winter semester)?” two participants investigated how many students are currently enrolled at this specific university to get an idea about how many freshmen there might be and how many people might apply there. This “meta‐strategy” appears to help them to even find a possible solution approach in the first place, to then apply the strategies listed above to (try to) calculate a solution. Exploratory information search thus occurred quite often, that is, 46 times over all trials across all participants.

Importantly, the protocols also reveal that when participants were unable to find an appropriate substitution or decomposition for a question, they were often stuck and reverted to gut‐feeling guesses. They were unable to answer, just retyped slight variations of the question into Google Search, or they simply guessed. Overall, this happened for their final answers 17 times (four of them remained unanswered, 13 were plain guesses). Furthermore, participants across all trials guessed 50 times during the solution process while working on subquestions. They also often indicated that their current strategy was not the best, and they wished they had a better idea. Sometimes, they would just stick with their current approaches because they did not know what else to do. At other times, participants switched their strategies within a trial but got stuck with the new one as well. Overall, this occurred 41 times across all trials. For example, participants mentioned “Well, I'm a little ‐ how should I say ‐ lost.” or said “This doesn't seem right, but I don't know what else to do.” referring to repeatedly failing to generate further ideas or approaches on how to get a better answer other than their first one.

#### 2.2.2 Analysis of performance during guesstimation

In addition to the strategies we identified and examined from the think‐aloud protocols, we also collected all participants' quantitative answers. In particular, we analyzed whether there are significant differences in performance between their first gut‐feeling answers and the deliberated, second responses. For this analysis, we excluded the unanswered trials and thus analyzed only the remaining 54 trials.

Since guesstimation is a “back‐of‐the‐envelope” calculation, which often aims to produce an answer in the “right ballpark,” that is, within one order of magnitude (Anderson & Sherman, 2010), we defined the response error as the $\log_{10}$ ratio of the response to the true value. A perfect response has a value of 0 and values greater than 1 or smaller than –1 mean that the participant was off by a factor of 10, that is, one order of magnitude. The mean response error for the gut‐feeling answers was –1.03 (*SD* = 1.59) and 0.14 (*SD* = 2.25) for the deliberated answers over all participants. Overall, our participants underestimated the value in 81.4% of the trials in their gut‐feeling answers and 55.5% of the time in their deliberated answers. We found a significant difference between the log‐ratios for the gut‐feeling and deliberated answers with a paired *t*‐test (*p* < .001) and a Wilcoxon signed rank test (*p* <.001). Comparing the absolute values of the $\log_{10}$ ratios did not reveal a significant difference (paired *t*‐test *p* = .67), with the mean for the gut‐feeling answers being 1.56 (*SD* = 1.06) and 1.46 (*SD* = 1.71) for the deliberated answers.

Since we had the think‐aloud protocols in addition to the quantitative data for all participants, we were able to make use of having this rich additional context to examine the answers more closely. We thus realized that a few aspects of the analysis can be improved. Specifically, the protocols revealed that one of the questions (Q5 in Table 1) we posed was ambiguous and could rightfully be understood in two different ways. The question was aimed at finding the overall number of movies that all Oscar winners for Best Actress were in, but some participants understood the question as the average number of movies one actress who won the Oscar for Best Actress is in during her career. In the German phrasing of the question, it was possible to misunderstand the question in this way. Since this was an error in the experimental stimulus, but we knew from the protocols which participants answered the question in which way, we calculated the resulting error of their answers correspondingly: for six participants, the true value was the average number of movies per actress, and for three, it was the overall number of movies of all Oscar winning actresses (one participant did not answer; this is the one question that remained completely unanswered both in the gut‐feeling and deliberated trials). As we knew both ground truth values, we adjusted our scoring in those cases accordingly. We also adjusted the scoring for these participants in their gut‐feeling answers. In addition, since we could retrace the steps of our participants for each question, we discovered another misunderstanding of some participants: for the question “How many minutes of TV was watched in Belarus per person in 2020?” the part “per person” was overlooked. Some participants thus calculated the right number, but for the overall population of Belarus and not per person. Thus, we corrected this error. We only corrected mistakes such as these if it was possible to clearly understand what the participants wanted to do, for example, if they simply forgot to complete a calculation they stated and planned to do in the beginning. We did not change any answers if we were unsure about what their solution idea was for what they calculated. This means, we made sure to only correct answers when we had enough explicit information in the data about the intended solution process of the participant. Lastly, we removed simple calculation errors where participants talked about the right way to calculate the answer but made a typo when using the calculator.

**Fig. 2:**
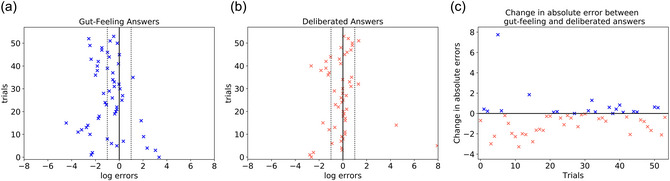
(a) and (b) $\log_{10}$ ratios of the participants' responses for the two parts of a trial. In both plots, the dotted line highlights where values are off by one order of magnitude, that is, within the dotted line, participants are “in the right ballpark.” Note that the data in both plots are adjusted as described in Section 2.2.2. (a) $\log_{10}$ ratios for the gut‐feeling responses in the first part of each trial. (b) $\log_{10}$ ratios of the participants' deliberated responses in the second part of each trial. (c) Change in absolute $\log_{10}$ ratios between gut‐feeling and deliberated answers across all participants and trials. Negative values indicate a reduction in error, that is, an improvement in the deliberated answers compared to the gut‐feeling ones. Blue values indicate either no change (some values are 0) or a decrease in performance (increase in error) in the deliberated, as opposed to the first gut‐feeling answer.

After cleaning the data with the aforementioned corrections and adjustments, our analysis shows a mean response error for the gut‐feeling answers of –0.81 (*SD* = 1.53) and deliberated answers of –0.07 (*SD* = 1.59) over all participants. Now, the participants underestimated the values in 79.6% of cases for the gut‐feeling answers and in 53.7% for the deliberated answers. We found a significant difference between the log‐ratios for the gut‐feeling and deliberated answers with a paired *t*‐test (*p* = .01) and a Wilcoxon signed rank test (*p* = .002). The $\log_{10}$ ratio responses are plotted for all trials for both the corrected gut‐feeling and deliberated answer over all participants in Fig. 2a and b, respectively. When examining the absolute values of the $\log_{10}$ ratios, this now also revealed a significant difference (paired *t*‐test *p* = .03). We evaluate the differences in the absolute values of the $\log_{10}$ ratios and find improved performance in 62.9% of deliberated answers compared to the gut‐feeling ones, which is a statistically significant improvement in performance in the deliberation part of the trial as compared to the gut‐feeling answers after the data was cleaned (binomial test *p*‐value = .02). These changes in the absolute errors between the gut‐feeling and deliberated answers are visualized in Fig. 2c, with the pink crosses indicating a reduction in the error, that is, an improvement for the deliberated answers and the blue crosses indicating no improvement or decrease in performance. The outliers where participants clearly overestimated the values (log error > 4) and that are clearly visible in Fig. 2b can be explained with the think‐aloud protocols. They show that in those trials, participants had extremely complicated strategies that either just did not lead to the desired value at all or would have required way more time and, therefore, led to guesses in the end. Additionally, in these data, we find a weak correlation (Spearman Rank Correlation Coefficient = .26) between the absolute errors of the gut‐feeling and the deliberated answers, but this correlation is not statistically significant (*p* = .06). This indicates that there could be some association between the performance in the gut‐feeling and deliberated parts of the trial, but it is not strong enough for either of the answer performances to be predictive of the other.

Lastly, the statements in our questionnaire at the end of the trials measured how our participants perceived their performance on a 5‐point Likert scale. We asked participants to indicate their agreement to statements regarding whether they felt like they handled the questions well and whether they knew how to best approach the questions (with 1 corresponding to completely disagree and 5 to completely agree). When we asked if they knew from the start how to approach a question, they indicated “somewhat disagree” on average, that is, the mean value was 2.5 (*SD* = 1.02), which indicates that they mostly felt like they did not have a successful strategy right from the beginning. This is in line with what we observed in most participants' think‐aloud protocols. Furthermore, asking participants to indicate their agreement about statements for their gut‐feeling answers, that is, “I think that my gut‐feeling answers were good estimates.” revealed a mean score of 1.6 (*SD* = 0.66), while the same statement about their deliberated answers had a mean of 3.2 (*SD* = 1.077). This indicates that participants, while not completely sure, considered their deliberated answers as better than their gut‐feeling estimates. All other items of the final questionnaire alongside the average value of answers are shown in Table 3.

**Table 3:** All items from the questionnaire at the end of Experiment 1 with the average answers and their standard deviations

Questionnaire item	Responses
I was able to handle the questions well right from the start.	Mean = 2.3; *SD* = 0.9
I knew right from the start how best to approach the answering of the questions.	Mean = 2.5; *SD* = 1.0
It was easy for to provide the quick answers.	Mean = 1.9; *SD* = 0.7
I had the feeling that I could already give good estimates for the quick answers.	Mean = 1.6; *SD* = 0.6
I had the feeling that I could give good estimates for the deliberated answers when I had more time and tools.	Mean = 3.2; *SD* = 1.0
I have often changed my estimates from the answers I gave in the quick part compared to the deliberated answers.	Mean = 3.7; *SD* = 0.9
I would have liked to have more tools.	Mean = 3.7; *SD* = 1.1
I would have liked advice and support during the answering and decision‐making process.	Mean = 2.9; *SD* = 1.0

*Note*. Participants indicated their answers on a 5‐point Likert scale with 1 corresponding to “completely disagree” and 5 to “completely agree.” Please note that all items are translated from German into English.

### 2.3 Discussion

While our participants were generally able to solve the guesstimation problems reasonably well, that is, mostly within one order of magnitude, the think‐aloud protocols still revealed some limitations and impasses. One of the most prominent observations in the data was that participants sometimes “got stuck.” This occurred when they were unable to brainstorm new approaches or decompositions. In these situations, participants often expressed that their current approach was probably not the best. When this was the case, they often gave another gut‐feeling response that was slightly more informed than their first answer for each trial, because they collected some more general information about different aspects of the question. For example, for the question “How many smartphone users are there in Dhaka?” participants often did not know where Dhaka was located and how many people lived there when they initially entered their gut‐feeling answers. Once they were able to research this information, they already improved their answers, even though their deliberation only led to adjusting for the population number. For example, one participant guessed 10,000 in the first part of the trial but when they found out that millions of people live in Dhaka, they improved their answers by increasing this number.

When comparing the quantitative answers that participants gave as their gut‐feeling as opposed to their deliberated answers, there is a significant difference in their errors. Overall, the difference in the absolute error indicates that participants improved their performance in their deliberated answers compared to the gut‐feeling ones. In the gut‐feeling answers, our participants generally underestimated the values more than with their deliberated answers. Therefore, while the deliberation process does not work perfectly and outliers can occur, participants still performed better in the second, deliberative part of each trial as compared to their initial gut‐feeling answers.

In contrast to our findings, some work (Bago & De Neys, 2019; Raoelison, Thompson, & De Neys, 2020) suggests that people who perform well in deliberative reasoning tasks are usually “smart intuitors.” This means that in a two‐response paradigm (Thompson, Turner, & Pennycook, 2011), where they first provide fast intuitive responses and then deliberate, they have already correct or better gut‐feeling responses to start with. These studies thus indicate no significant improvement through deliberation in their tasks, but rather argue that those who perform well have good intuition that influences their performance more than the effect of deliberation. However, the tasks in these studies are, for example, the bat‐and‐ball or base‐rate‐neglect problems that are often incorrectly answered and where all information is already provided for the first response. It is important to note that in our study, gut‐feeling responses were given within 30 s and participants only focused on the task at hand. This is in stark contrast to the extremely fast gut‐feeling responses used by Bago and De Neys (2019), where participants only had 4 s, and there was a secondary load task. Bago and De Neys (2019) purposely used such a challenging task to minimize any possible deliberation in participants' initial responses. In our task, pilot testing showed that 30 s were appropriate to ensure that participants had enough time to read and comprehend the question before giving their answers. Hence, our gut‐feeling responses may not be completely free of deliberation but participants did not have a lot of time to think about an answer either—they certainly did not use a calculator or Google in that time. Generally, as gut‐feeling responses in our setting were not too far off either, we believe that our results do not contradict those by, for example, Bago and De Neys (2019), as they find that “[w]henever people manage to give the correct […] answer as their final response after deliberation, they often already selected this answer as their initial response when possible deliberation was experimentally minimized.” Although we show that in a guesstimation task, gut‐feeling responses may not be far off from the correct response, they still improve through deliberation. However, in contrast to, for example, the bat‐and‐ball task, guesstimation tasks are not just about finding the “correct” answer, but rather a “better” one. Participants in our task do not have all necessary information available through the task instructions and finding new, relevant information is an important part of the deliberation process. Thus, when using guesstimation(‐like) tasks, where the reasoning relies on problem decomposition (Tetlock & Gardner, 2015; Weinstein, 2012), creativity (Okamoto, 2022; Wessels, 2014), and often on uncertain information, we find that deliberation significantly improves the answers of participants—even if their initial gut‐feelings are not too far off as well. Our findings align well with other work (Mellers et al., 2015, 2015; Tetlock & Gardner, 2015) that shows that those who perform best in guesstimation‐like tasks, such as forecasting, are those who deliberate more and more systematically.

Lastly, analyzing the answers to the questionnaire at the end of the experiment revealed that our participants had doubts about the quality of their responses. Therefore, in the next experiment, we examine not only their performance further, but also investigate their confidence about their answers more systematically.

## 3 Experiment 2: Confidence judgments for guesstimation

In Experiment 2, participants also answered guesstimation questions, but in addition to giving an estimate, they also had to specify their uncertainty. They provided both by visually adjusting a normal distribution on a response scale.

### 3.1 Methods

We conducted an online study with 48 participants. They all were university students aged between 18 and 30 years (30 females, 18 males). The study was approved by the local ethics board and all participants provided informed consent. The study was conducted in their native language (German).

We investigate whether the confidence indicated with their answers was well‐calibrated, that is, whether the participants' confidence was higher when their answers were closer to the true value and lower when they were further away from it. We used the same web interface as in the previous study (with fields for sending queries to Google, taking notes, and a calculator), which is shown in A.2 in Fig. 5. We used some of the same questions (correcting any misunderstandings in their phrasing, e.g., for Q5) but also added new ones. This was necessary because the answers to some of the questions used in Experiment 1 could now be found online at the time we conducted Experiment 2. An overview of the questions used in Experiment 2 is given in Table 1. In contrast to the previous study, this study was conducted online due to COVID‐19 lock‐downs at the time. We did not collect any screen or audio recordings, but there was a short on‐boarding video call at the beginning. During this call, the experimenter explained the instructions on a test screen with a test question. All functionalities, as well as the aim of the study, were explained. As before, the participants were instructed to give the best possible answer within a time limit of 8 min, and the experimenter answered any remaining questions. In contrast to Experiment 1, we did not ask for a gut‐feeling answer first because our main interest was whether their deliberated answers are well‐calibrated. Once the setup, explanation, and test trial were completed, the video call was ended, and the participants completed all trials by themselves (without any supervision).

The main difference in the experimental design and instructions was the way in which the participants provided their answers. They gave each answer in the form of a normal distribution. They were instructed to type in the answer that they thought was most likely as the mean of the distribution, and then had to adjust the shape of the distribution in order to indicate how certain they were about it.

In a drop‐down menu, participants chose a scale (e.g., size unit of a 100 if they want to enter a mean in that order of magnitude). The chosen unit is then shown on the x‐axis as the scale immediately. Participants then enter their mean (e.g., for the example before, they could enter 3.7 if they want their mean to be 370).

A non‐normalized normal distribution was displayed on top of a ruler with tick marks according to the chosen scale. The shape of the normal distribution was then adjusted by using a slider. We use this method to ensure that the display of the scale and the entry of the distribution is the same across trials, even if the numbers needed as answers vary widely across the different guesstimation questions (e.g., Q1 in Table 1 requires an answer within hundreds, while Q6 requires an answer in millions). Because the responses required different scales, ideally, participants should report their estimates and uncertainties on a log scale. However, in pilot testing, we found this to be too hard for participants to understand and, therefore, opted for having them report a normal distribution on a linear scale. Participants were told that the more certain they are about their answers, the tighter the distribution, and the less certain they are, the broader it should be. By eliciting the participants' answer in this way, we collected both the indicated mean and the indicated standard deviation for each final answer of every single trial. As before, we additionally collected their Google search terms, notes, and calculations.

### 3.2 Results

**Fig. 3:**
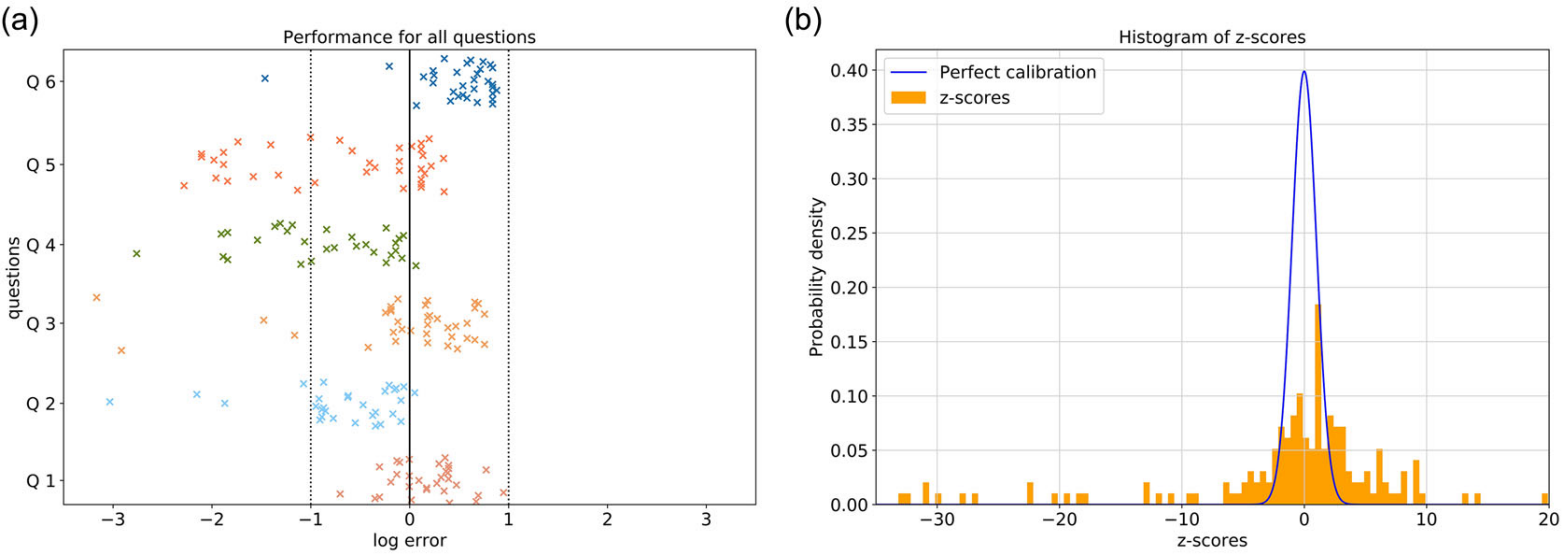
(a) $\log_{10}$ ratios of the responses to the true values for all guesstimation questions. The numbers for each question are corresponding to the questions in Table 1, for example, Q1 refers to the response errors when participants answered the question about the car sharing vehicles. (b) In the orange histogram, the z‐scores of the participants' answers over all trials are shown. If they were perfectly calibrated, these would be distributed standard normally, but this is not the case as can be seen by comparison with the blue standard normal distribution. Note that the x‐axis is cut‐off (on the left side) for better visibility, and some bins remain outside its bounds.

Just like in Experiment 1, we used the $\log_{10}$ ratio of the response to the true value as the error. In a first step, we only analyzed the mean of the normal distribution that the participants provided as their answers. The errors of these responses are shown in Fig. 3a for each question for all trials. The mean error was –0.27 (*SD* = 0.51) across all participants. Overall, 58.3% of the participants' answers underestimate the true value, but some questions are much more prone to underestimation than others. On average, participants completed a trial and gave their answers in 4 min and 39 s (mean = 279.2 s, *SD* = 95.3 s). The distribution over all participants' completion times is shown in the Appendix (see A.3 Fig. 6b).

We analyzed how the participants were calibrated by looking at the means and the standard deviations of the normal distributions that we elicited from them. If a participant's estimate is close to the true value and the participant knows it, the indicated shape for the normal distribution should be narrow. But if the estimate is far off from the true value, the confidence should be low and thus the shape of the distribution should be broad. To assess participants' calibration, we z‐scored their responses,

(1) $$z_{pq}=\left(m_{pq}-x_{q}\right)/s_{pq},$$

where $z_{pq}$ is the z‐score of participant p for question q, $m_{pq}$ is the mean of the normal distribution that we elicited from participant p for question q, $x_{q}$ denotes the true value for the question, and $s_{pq}$ is the standard deviation of the elicited distribution.

Thus, the z‐scores are the differences between the participants' answers and the true values, measured in the standard deviations that they provided to indicate their certainty.

While it would be interesting to investigate each participant's confidence, we do not have enough data points to determine individual calibration as there are only six trials per participant at most. However, we can examine the entire sample, and if all participants were perfectly calibrated in all trials, then the z‐scores should follow a standard normal distribution. In Fig. 3b, we show the z‐scores of the participants' answers over all trials as a histogram in orange. The x‐axis is cut off on the left side for better visibility, and some bins remain outside its bounds. It is clearly visible that the participants' responses do not follow the standard normal distribution shown in blue. Nevertheless, we tested for normality with a Shapiro–Wilk test as well, and the *p*‐value is smaller than .01, meaning that the z‐scored responses are significantly different from a normal distribution and, hence, the participants are not perfectly calibrated.

**Fig. 4:**
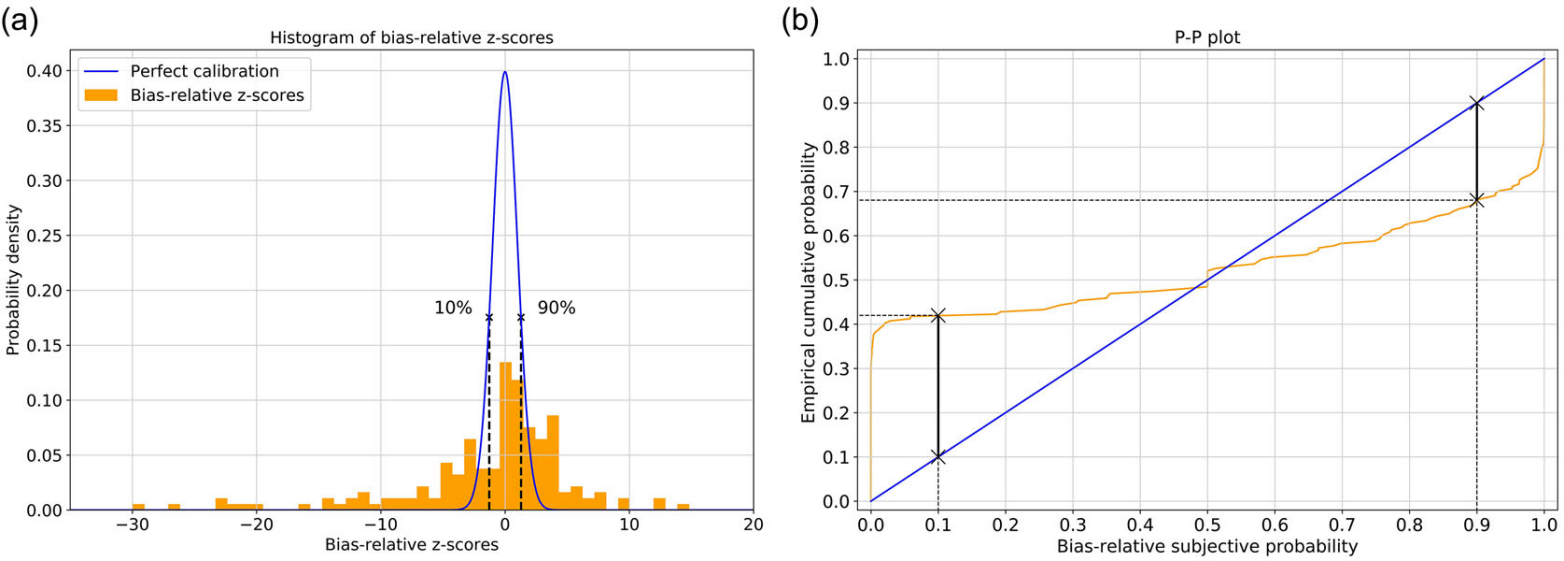
(a) In the orange histogram, the bias‐relative z‐scores of the participants' answers over all trials are shown. If they were perfectly calibrated, these would be distributed normally, but as is shown with the blue standard normal distribution, this was not the case. Note that the x‐axis is cut‐off (on the left side) for better visibility, and some bins remain outside its bounds. (b) Corresponding Probability‐Probability plot for assessing how close the participants' accuracy in their answers is to their indicated confidence. Both plots show the answers and confidence measures for all participants over all trials.

**Fig. 5:**
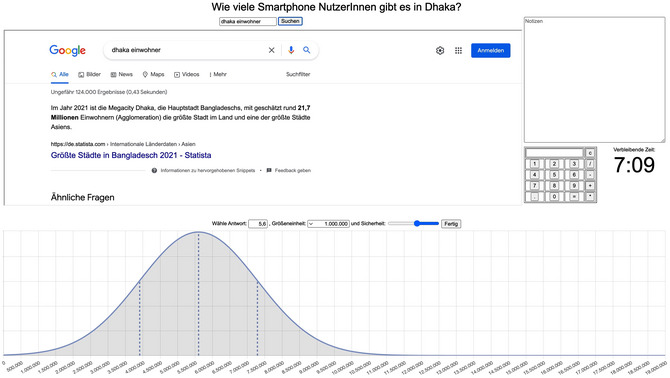
Web interface for experiments. We always provide the search bar for searching all information on Google. Once a search is typed into the search bar of our experiment, the search keys are saved. All results related to the search terms on Google are displayed in a window within the interface. In addition, on the right‐hand side, we provide a notepad and a calculator. In Experiment 1, participants only had a text field to insert a numerical value as their final answer. In Experiment 2, their answer distribution was indicated and displayed like shown here at the bottom.

**Fig. 6:**
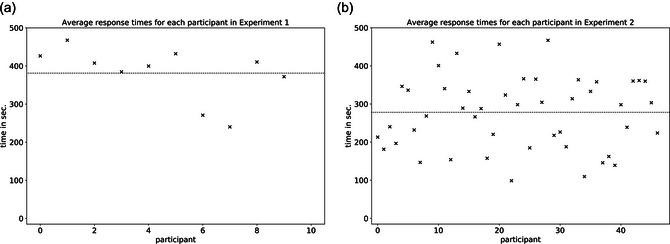
Average response times for all participants in Experiment 1 (deliberated part) and Experiment 2. (a) The 10 participants, on average, gave their answers in 6 min and 21 s, that is, 381 s. This average is marked with the dotted line. (b) The 47 participants, on average, gave their answers in 4 min and 39 s, that is, 278 s. This average is marked with the dotted line.

However, we did not expect all participants to be perfectly calibrated. We can see in Fig. 3a that different questions have different response biases. While for some questions, participants systematically overestimate the true value, for others, they underestimate it. Participants, obviously, are not aware of these systematic biases because, otherwise, they would correct for them in their deliberation process. Hence, some of the miscalibration that we have described thus far can be explained by these biases. However, while participants cannot judge their uncertainty relative to the true value, they might be able to relative to the bias that all estimates display. We, therefore, define a new measure

(2) $$z_{pq}^{*}=\left(m_{pq}-M_{q}\right)/s_{pq},$$

that is, again a z‐score, with the only difference being that we score against the median response $M_{q}$ over all participants instead of the true value $x_{q}$. We refer to this second z‐score as bias‐relative to distinguish it from the truth‐relative z‐score. We present the same data as before but scored against the bias that all participants have in Fig. 4a. Again, the x‐axis is cut‐off (on the left side) for better visibility and some bins remain outside its bounds. It is clearly visible again and confirmed by a Shapiro–Wilk test (*p*‐value < .01) that the z‐scores of participants are not normally distributed, and they are not perfectly calibrated.

We analyze the data further with respect to the overall relation between the indicated confidence and the performance of the participants. We visualize this in a Probability‐to‐Probability (P‐P) plot in Fig. 4b. P‐P plots compare two cumulative distribution functions (CDFs). Specifically, one can visually compare an empirical to a theoretical distribution. In our case, if we assume perfect bias‐relative calibration of all participants, we should get a standard normally distributed CDF, and we can compare this shape to the empirical CDF. For each z‐score, we can ask what proportion of the participants' z‐scores should be smaller and compare this proportion against the empirical proportion of how many z‐scores are actually smaller. This gives the P‐P‐plot in Fig. 4b, where the blue main diagonal shows the prediction according to the participants' confidence and the orange line shows the reality.

As an example, let us look at the bias‐relative z‐score in Fig. 4a, where the blue standard normal distribution predicts that according to the participants' confidence, if they were perfectly calibrated, 10% of their z‐scores should be smaller than this value. In reality, 42% of the orange distribution lies to the left of this value, as can be seen in Fig. 4b. Similarly, if we look at the z‐score for which 90% of the participants z‐scores should be smaller, only 68% actually are.

### 3.3 Discussion

The data in Fig. 4b relating the participants' bias‐relative subjective probability to the empirical cumulative probability indicate overconfidence and overextremity (Koehler, Brenner, & Griffin, 2002; Koehler & Harvey, 2008). Note that Fig. 4b seems similar to the usual confidence plots for binary decisions, where confidence scores on one axis are plotted against the actual performance on the other axis. While the meaning is similar here as well, showing overconfidence in our participants' answers, our plot is a P‐P plot based on the bias‐relative z‐scores. We do not use the true value of the answer for the questions, because this would not allow to disentangle any deviations resulting from either participants' bias or variance. It is impossible for the participants to know their own bias, and if they did, they would not have it. Thus, we use the bias‐relative z‐scores in this analysis. We, too, cannot know each participants' bias from a single response, but we can estimate biases that are shared across participants. The remaining variance across participants is still a lot bigger than the variance that participants reported when they indicated their individual certainty.

Similar to work on other judgment and reasoning tasks (Chabris & Simons, 2009), we also find overconfidence (or overprecision, i.e., confidence intervals are too narrow (Moore & Healy, 2008)).

Like all other probability elicitation methods, our method comes with certain caveats. In pre‐tests, we tried variations (e.g., testing log‐normal distributions, logarithmic scales, and normalized and non‐normalized distributions); however, all of them lead to different difficulties in understanding on the participants' side. Using a non‐normalized version of the normal distribution seemed to be most intuitive for most participants. It is, however, possible that some of the participants would have preferred a skewed log‐normal distribution or a log‐scale, as they had difficulties being precise in matching their confidence to the broadness of the distribution (especially for questions that required very large numbers). In general, the results of any elicitation method should not be interpreted as an unbiased and noise‐free measurement of a subjective probability. Hence, it is very likely that some of the overconfidence that we observe is due to individual biases and additional variance that is introduced by our elicitation method. Our elicitation method differs from other methods like eliciting percentages, where the indicated uncertainty can depend on the specific phrasing of the question (Løhre & Teigen, 2016), or Likert‐scales, which were often used in previous work (Bennett et al., 2018; Silver et al., 2021). While these methods seem simpler in some sense, respondents may still not understand the meaning of the response options or may interpret them differently. While we believe that our elicitation method and the corresponding analysis provide a quick and easy way to assess calibration in guesstimation problems, future work should explore more complex methods (see, e.g., O'Hagan et al., 2006).

While there could be many reasons for miscalibration in judgment tasks (Griffin & Brenner, 2004), a factor that might have influenced the confidence of our participants could be their access to the internet. Using the internet was useful to test guesstimation in a realistic setting compared to previous work (see, e.g., Gomilsek et al., 2024) and is also an essential part of real‐world applications such as (geo‐)political forecasting (Tetlock & Gardner, 2015). The use of the internet in such deliberative tasks can lead to better answers in deliberative tasks through access to facts and information, but people also significantly overestimate their performance in many tasks when using the internet (Pieschl, 2021). This might have affected the calibration of our participants as well.

## 4 General discussion

In this paper, we propose guesstimation problems as an interesting test‐bed for investigating human deliberative judgments. Not only can they be studied in the lab and performance can be scored quantitatively (cf. Figs. 2 and 3a), they are also not just toy problems either: They are challenging and have many real‐world applications, for example, forecasting for business proposals or intelligence reports. They are also used in education, especially in math classes, to teach general problem‐solving skills (Albarracín & Gorgorió, 2012, 2015; Ärlebäck & Albarracín, 2019b; Ärlebäck & Albarracín, 2024). Therefore, we empirically investigated how humans solve such guesstimation problems, how well they perform in this task, and whether they are well‐calibrated in their confidence about their answers. In order to do this, we designed guesstimation questions that we can score quantitatively. In contrast to other studies (e.g., Gomilsek et al., 2024), we provided participants with plenty of tools and access to the internet. While this makes it harder to compile a large set of quantitative questions that the experimenter, but not the participant, knows the answer to, such a design is arguably much more representative of real‐world guesstimation.

With our first experiment and through think‐aloud data, we gained a deeper understanding about the strategies that participants used to answer guesstimation questions. We not only identified strategies from previous work within the protocols (Abourbih, 2009; Abourbih et al., 2010; Paritosh & Forbus, 2004; Paritosh and Forbus, 2005), but also discovered additional ones. Specifically, we find that besides the known strategies from previous work, participants used fudge factors and ontological similarity (a combination of strategies from Paritosh and Forbus (2005) that are usually applied *together*). Furthermore, we often observed the “meta‐strategy” of exploratory information search, which was used to find a solution approach in the first place. While existing work (Abourbih, 2009; Abourbih et al., 2010) relied on best‐practice guides and examples for guesstimation (Weinstein, 2012; Weinstein & Adam, 2008), here, we empirically examined the specific steps humans take to solve guesstimation problems in a variety of domains and topics.

It might be possible to improve existing systems, such as BotE‐solver or GORT, by including the new strategies that we identified here, for example, by combining the ontology and similarity strategies of Paritosh and Forbus (2005) into the ontological similarity strategy. Including the meta‐strategy of information search might be useful as well. It was shown recently that current large language models perform better in reasoning tasks (which can include guesstimation), when they are prompted to first generate general information and facts about the subject matter of the question at hand (Liu et al., 2022). Of course, it might not always be straightforward to implement these additional strategies. Fudge factors are a good example. However, it is interesting that participants have some intuitions about the numbers they use and that these intuitions can improve their answers. The intuitions seem to be informed by the deliberation process, even though participants do not give explicit reasons for applying fudge factors. However, participants seem to be keeping track of the (cumulative) inaccuracies in their partial estimates and try to correct for them. This approach would also be beneficial for artificial systems.

We designed our studies such that we were able to empirically examine the quality of the answers. We find that humans can solve guesstimation problems reasonably well, especially when they get the chance to deliberate. While even gut‐feeling answers (given in 30 s or less) were already decent (see Fig. 2a), they improved further when given deliberation time (see Fig. 2b). The absolute error also decreased for participants' deliberative answers compared to the gut‐feeling ones, as shown in Fig. 2c. This aligns well with the findings in previous work on forecasting tasks (Mellers et al., 2015, 2015; Tetlock & Gardner, 2015). However, these works usually target binary yes/no questions, which can be analyzed with Brier scores. Here, we tackle the case of participants making quantitative judgments. Deliberation thus did not only improve participants' answers in our experiment, but instructing deliberation by considering (more) decompositions of the problem at hand was also found to be useful by others (Gomilsek et al., 2024).

Furthermore, we examined how well‐calibrated the participants were in our second study. We asked them to indicate their answers as the mean and adjust the standard deviation of a normal distribution based on whether they were sure (i.e., a narrow distribution) or unsure (i.e., a broad distribution). This elicitation method for participants' confidence in guesstimation allowed us to investigate calibration systematically. Other common elicitation methods are percentages (Løhre & Teigen, 2016), confidence bounds (Gomilsek et al., 2024), or Likert‐scales (Ais, Zylberberg, Barttfeld, & Sigman, 2016; Lee & Daunizeau, 2020). The advantage of eliciting normal distributions as we did here, however, is that it is quick, but calibration can still be assessed in a straightforward way by looking at P‐P‐plots in lieu of the calibration plots that are used for binary events.

The results show that participants are overconfident (see Fig. 4b), which is well‐aligned with previous work for other reasoning and deliberative judgment tasks (Gomilsek et al., 2024). In addition to identifying the underlying solution steps and approaches, investigating calibration in guesstimation‐like tasks is one of the main contributions of this paper, as it is a crucial factor to consider when trying to improve estimates. This is true for group settings, where an answer is generated collectively (Bennett et al., 2018; Silver et al., 2021), but also when deliberating individually (Gomilsek et al., 2024). Despite their calibration being far from perfect, participants generally perform quite well in our guesstimation tasks. Inspecting the spread of the error responses in Fig. 3a indicates that there seems to be a difference in how difficult the questions were. While some questions reveal smaller deviations, that is, less severe errors (such as Q1 and Q6), there are others that show more severe errors (such as Q4 and Q5).

Interestingly, often the participants did not use the full deliberation time they had available to give their answers, with the mean response time being around 4 1/2 min. This means our participants, on average, would have had more time to deliberate, but they chose not to use it. This might also be due to their overconfidence.

When solving guesstimation problems, participants had many impasses where they did not know how to continue or change their approach, which became obvious in the think‐aloud protocols of Experiment 1.

One way to improve judgments thus could be to reduce the number of impasses during deliberation. In a companion paper (Salikutluk, Koert, & Jäkel, 2023), we specifically targeted the main reason why people get stuck and cannot decompose questions further: They fail to apply the ontological similarity strategy described above. We aimed to support humans in generating such transformations with a brainstorming tool that exploited recent advances in artificial intelligence (AI). Specifically, we prompted a large language model (LLM) with the successful transformations from think‐aloud protocols collected from our participants during guesstimation and showed that the LLM was able to generate reasonable and human‐like semantic transformations. Thus, we created an LLM‐based tool capable of brainstorming such transformations when humans reached impasses during guesstimation. While the tool tested in our study did not improve the performance significantly, we still see a lot of potential for AI to support human guesstimation. In fact, recent work on LLMs shows that “tree‐of‐thought” prompting, which is similar to the decompositions we find in Experiment 1 and show in Fig. 1, improves performance on complex tasks, for example, in mathematical reasoning, creative writing, or crosswords (Long, 2023; Yao et al., 2024). It has also been shown that iterated decomposition with a human‐in‐the‐loop approach can improve LLMs in scientific reasoning tasks (Reppert et al., 2023). Overall, these studies point toward the potential for using empirical insights on human problem‐solving, such as ours, to improve AI systems. In particular, our results might be used to design interactive AI systems that can solve guesstimation problems better together with humans. Such a system will be especially useful if the goal is to not just generate single solutions to guesstimation problems but rather come up with a variety of estimates: Working through several solutions to check and refine previous (partial) answers instead of just using the first one as the final estimate has been found to lead to the best performance (Gomilsek et al., 2024; Tetlock & Gardner, 2015), and AI systems could provide ideas for further approaches, decompositions, or transformation within this process.

To conclude, guesstimation problems are a suitable test‐bed to understand and investigate human deliberative judgments. Exploring both qualitatively and quantitatively how humans solve such problems might help foster (more) creativity (Okamoto, 2022) and (general) problem‐solving skills in the classroom (Albarracín & Gorgorió, 2014, 2015; Ärlebäck & Albarracín, 2019b). Furthermore, these insights are also relevant for improving forecasting and decision‐making in high‐stakes real‐world scenarios, such as in (geo‐)political judgments (Abeliuk, Benjamin, Morstatter, & Galstyan, 2020; Doyle et al., 2014; Auswärtiges Amt, 2021; Roff, 2020). While there has long been a desire in different disciplines to try and improve real‐world decision‐making (like, e.g., forecasting but also other such tasks) by basing it on quantitative analyses instead of fallible human judgments (Meehl, 1956), there are many areas where human judgment is indispensable (McAndrew, Wattanachit, Gibson, & Reich, 2021), even if it could be further enhanced by quantitative tools. An improved understanding of how people solve guesstimation problems can thus help us create AI tools that are well‐integrated with the strategies that are described in this paper (Salikutluk et al., 2023). Such a human‐centered approach (Shneiderman, 2022) promises to support and benefit human analysts and decision‐makers, instead of trying to replace them. This could allow for their strengths and those of the tools to be complementary (Rastogi, Leqi, Holstein, & Heidari, 2023), which was already shown to be promising in other settings (Holstein & Aleven, 2021) and tasks (Steyvers, Tejeda, Kerrigan, & Smyth, 2022).

## Open Research Badges

This article has earned Open Materials badges. Materials is available at https://osf.io/6nafs/?view_only=7bd54030a62d4f0e84aa6aff39ea1b86.

## Appendix A

### A.1 . Instructions for Experiment 1

The following instructions were shown to the participants in Experiment 1. After they read through them, they completed the training round. After the training round, they could read the instructions again, ask any question to the instructor, or start the experiment.

The following study focuses on estimation tasks. An example for the estimation task in question is the following: **“How many grains of rice fit in a coffee cup?”**

These are therefore tasks and questions to which there is no exact and correct solution or answer. Rather, the answers depend on how you approach the questions and how you answer them. The answers to these estimation questions must always be numerical values. Your task will be to answer these questions as best you can, thinking out loud. This means that you should speak all your thoughts out loud so that we can understand your approach. Please try to give the most accurate and best possible answer that you think is as realistic or as close as possible to the unknown real value.

There will always be two parts to a question. In the first part, you should give an answer quickly and “based on your gut feeling”. In the second part, you will then have time to deliberate and research the question in more detail and work out an answer. The interface and all relevant parts are now shown using the example above. Familiarize yourself with the overview of the interface and the functionality of the individual parts.

*[Showing interface of part 1]* First you are presented with a question and asked to quickly enter an answer into the corresponding field. Quickly here means that you will have 30 seconds as soon as the question is displayed. The point here is not that you provide a fully thought‐out answer, but that you initially answer, “from your gut feeling” and simply state quickly which number you would choose. You should enter an answer before the 30 seconds are up and finish this part by clicking on “Done”. If you do not do this, you will be given another 15 seconds to enter your answer. However, please make sure that you decide on an answer within the 30 seconds, enter it in the appropriate field and click “Done” to submit it. If no answer is given, the trial continues to the next part.

*[Showing interface of part 2]* After you have completed the first round, you will have time to deliberate and consider the question in more detail. You will be presented with the same question again, but this time you will have eight minutes to think about it and give an answer. Please remember to think out loud and speak your thoughts as unfiltered as possible the whole time. Please try to give the best possible answer, i.e. an answer that you think could be as close as possible to the (unknown) real value. You will be provided with a notepad, a calculator and an integrated Google search to gather information and work on the best possible answer. Please try to describe your thoughts that lead to your solution while using all these tools in any way you see fit.

Note on Google search: if you use it, it will open a small window within the page. However, please only use the search bar provided by the site for the entire duration of the experiment. Do not open any other browser tabs or windows, but only use the corresponding search engine. If you click on a page during your search and then want to return to the overview of search results, please do not use the browser's “Back” button. Instead, please click on “Search” again in the search bar provided.

Once you have decided on an answer and entered it, you can finally confirm it by clicking on “Done”. If you forget or do not manage to click on “Done” before the time runs out, but an answer has already been entered in the corresponding field, it will still be saved. After giving your deliberated answer as well, you are asked to fill out a short questionnaire about your approach and the process of finding and deciding on your answer. If you were unable to open a page, please indicate this in your answer to the question after the respective run. Once you completed the deliberated second part and fill in the questionnaire, the trial is completed. You will work through 6 trials overall in this way.

Now it's time for the practice round, in which you can test the two sub‐rounds and familiarize yourself with the task, the structure and the function. Please start thinking out loud and continue to do so in both parts and for all the following questions. Should you stop talking and expressing your thoughts the instructor will remind you to keep talking.

### A.2 . Interface for experiments

The interface shown in Fig. 5 is the interface used for Experiment 2. There, we instructed participant to indicate the most likely answer as the mean. To ensure that the display of the distribution stayed the same and was able to shown in a understandable way for our participants, they had first choose the corresponding scale needed for the answer and then insert the value that would lead to their answers. For example, if they wanted 5.6 million to be their (as shown in Fig. 5), they needed to choose the scale “1.000.000” (“Größeneinheit”), and then write 5.6 into the answer field (“Wähle Antwort:”). Since we asked participant to indicate a distribution, we then displayed a non‐normalized standard normal distribution around their indicated mean value, and with the slider, they then adjusted the width of the distribution according to their certainty (“Sicherheit”).

For Experiment 1, we used the same interface but without the distribution as the answer field. Instead, participants in Experiment 1 simply had a field to write the numerical value they wanted to indicate as their answers. Everything else remains the same on the interface.

### A.3 . Average response times of all participants in Experiments 1 and 2

Participants had 8 min for each trial available to deliberate and find the best possible answer in both Experiment 1 (deliberated part) and Experiment 2. The average response times are shown in Fig. 6.
